# Ginsenoside Rh2 Inhibits Glycolysis through the STAT3/c-MYC Axis in Non-Small-Cell Lung Cancer

**DOI:** 10.1155/2021/9715154

**Published:** 2021-09-25

**Authors:** Xiaodan Sun, Peiyan Zhao, Hui Li, Yan Liu, Tianming Wang, Ying Cheng

**Affiliations:** ^1^Postdoctoral Research Workstation, Jilin Cancer Hospital, Changchun 130012, China; ^2^Translational Cancer Research, Jilin Cancer Hospital, Changchun 130012, China; ^3^College of Traditional Chinese Medicine, Changchun University of Chinese Medicine, Changchun 130117, China; ^4^Department of Medical Thoracic Oncology, Jilin Cancer Hospital, Changchun 130012, China

## Abstract

Ginsenoside Rh2 (Rh2) is one of the pharmacologically active components of ginseng with an antitumor effect. However, its effect on non-small-cell lung cancer (NSCLC), especially on aerobic glycolysis, which plays a crucial role in the proliferation and progression of tumor cells, has not been characterized. Here, we demonstrated that Rh2 inhibited the proliferation and metastasis of NSCLC cells by promoting apoptosis and suppressing epithelial-mesenchymal transition, respectively. Notably, Rh2 exerted a glycolysis inhibition effect through regulating GLUT1, PKM2, and LDHA, which are key enzymes of the glycolysis process. Furthermore, the metabolic shift function of Rh2 was dependent on the STAT3/c-Myc axis in NSCLC. This novel regulatory role of Rh2 provides a new perspective for NSCLC treatment and highlights the potentiality of Rh2 to be used as a tumor energy blocker. The combination of Rh2 with an STAT3 or c-Myc inhibitor revealed a promising therapeutic approach for patients with NSCLC.

## 1. Introduction

Lung cancer, which remains the leading cause of cancer-related death, is a major public health concern in the world [1]. Approximately 85% cases of histological type are non-small-cell lung cancer (NSCLC) [2]. Traditional Chinese medicine is a treasure of traditional culture in China. The combination of traditional Chinese medicine and modern treatment has attracted great attention in overcoming NSCLC. However, the ambiguous drug target and limited fundamental research of Chinese medicine restrict its clinical application. Therefore, there is an urgent need to clarify the potential antitumor target or mechanism of Chinese medicine.

Ginsenoside Rh2 (Rh2) is one of the major active substances of Ginseng, which is one of the oldest and most widely known Chinese herbal medicines, first documented in Shen Nong's Materia Medica from the Han dynasty. Rh2, similar to other ginsenosides, possesses a wide spectrum of pharmaceutical activities, including improving immunity, enhancing memory ability, antidepressant, and cardiovascular protection, and Rh2 monomer has been used as a herb supplement termed JinXing Capsule in China since 2006, but emerging research has found that it has a potent antitumor property through various mechanisms, such as inhibiting proliferation, invasion, inducing apoptosis, triggering cell cycle arrest, and improving chemotherapy sensitivity of tumor cells [3]. In terms of structure, Rh2 can be divided into S-type and R-type configurations according to the stereospecificity of the hydroxyl group at carbon-20, among which 20(S)-Rh2 monomer was reported to play a major role in anticancer activities [4]. Thus, Rh2 refers specifically to 20(S)-Rh2 in this study.

The tumor microenvironment has been widely believed as an important factor for tumor cell proliferation, invasion and metastasis, and immune escape. Aerobic glycolysis, also termed as the Warburg effect, is a novel landmark of the tumor, which means that glycolysis is the main energy metabolism pathway regardless of the oxygen levels [5]. High-speed aerobic glycolysis of tumor cells could produce amounts of lactate, resulting in an acidic microenvironment [6]. Dysregulation of pH in cancer has been described as a “perfect storm” that promotes tumor cell survival and evasion of apoptosis and increases the invasive and migrative phenotype of tumor cells [7]. Therefore, inhibiting glycolysis and neutralizing the acidic microenvironment may become a potential antitumor scheme. However, the role of Rh2 on NSCLC glycolysis remains unclear. Therefore, this study intended to explore the effect and possible mechanism of Rh2 on human NSCLC cell lines, to provide a theoretical basis for the application of Rh2 in clinical practice.

## 2. Materials and Methods

### 2.1. Reagents

20 mg Ginsenoside Rh2 (Rh2, ALADDIN, Shanghai, China) was dissolved into 1 ml dimethyl sulfoxide (DMSO, Sigma-Aldrich, Germany) to make a final concentration of 20 mg/ml stock solution. Fetal bovine serum (FBS, Invitrogen, Carlsbad, CA, USA)-free RPMI-1640 medium was used to dilute the stock solution to the indicated working concentrations. The maximum final concentration of DMSO was under 0.1% for cell treatment.

### 2.2. Cell Culture and Transient Transfection

Human NSCLC cell lines A549 and H460 (Shanghai Cell Bank, Chinese Academy of Sciences, Shanghai, China) were cultured in RPMI-1640 medium with 10% FBS. The culture environment was 37°C and 5% CO_2_.

Lipofectamine 3000 reagent (Invitrogen, Carlsbad, CA) was used to perform transient transfection according to the manufacturer's instructions. Human c-Myc expression vector and empty vector (catalog number: POSE146072729, GeneChem, Shanghai, China) were used for c-Myc overexpression and negative control, respectively.

### 2.3. Cell Proliferation and Colony Formation Assays

Cell proliferation and colony formation assays were performed as described previously [8]. Briefly, Cell Counting Kit-8 (CCK-8, Beyotime, Shanghai, China) assay was used for cell proliferation. 5000 cells/well were seeded in a 96-well plate and treated with serial concentrations of Rh2 (0, 5, 10, 20, 40, and 80 *μ*g/ml) for an indicated time (24, 48, and 72 h). 10 *μ*l of CCK-8 reagent was mixed into each well, and the absorbance (OD) at 450 nm was measured using a microplate reader (CLARIOstar, BMG LABTECH, UK).

Cell viability = (Experimental group OD −blank well OD/Control group OD −blank well OD) × 100%. GraphPad Prism 8.0 software was used to calculate values indicating 50% inhibition of the surviving fraction (IC50).

For the colony formation assays, 500 cells/well were seeded in 6-well plates and treated with 0, 10, and 20 *μ*g/ml Rh2 for 14 days. The number of colonies containing more than 50 cells was counted by using a microscope.

### 2.4. Cell Apoptosis

The analysis of cell apoptosis was performed according to FITC Annexin V Apoptosis Detection Kit I's (BD Biosciences, USA) protocol. Briefly, after treated without or with 40 *μ*g/ml Rh2 for 48 h, the harvested cells were resuspended in 1 × Annexin V-binding buffer, stained with FITC-conjugated Annexin V and PI. Cell apoptosis was analyzed using a flow cytometer (FACSAria III, BD Biosciences, USA).

### 2.5. Cell Migration and Invasion

Wound healing assay was performed to measure the cell migration viability. NSCLC cells were plated in 6-well plates and wounded by a 250 *μ*l pipette tip when a confluent monolayer was formed. The cells were cultured in FBS-free medium in the presence or absence of 40 *μ*g/ml Rh2 for 24 h. Images of the same position of the wounded monolayer were obtained using a microscope. ImageJ software was used to measure wound width quantitatively.

Transwell assay was used to observe cell invasion viability. Transwell chamber (5 *μ*m pore size, Millipore, USA) was coated with 50 *μ*L Matrigel matrix gel (BD Biosciences) for 24 h. Then, 600 *μ*l of medium containing 10% FBS was added to the bottom of the chamber and 100 *μ*l of NSCLC cell suspensions at a density of 5 × 10^4^ cells/ml in FBS-free medium treated without or with 40 *μ*g/ml Rh2 was seeded into the upper chambers. After 24 h, the cells adhered to the upper surface of the transwell membranes were removed and wiped off with cotton swabs, while those on the lower surface were fixed with 4% paraformaldehyde and stained with 0.1% crystal violet. The invaded cells were photographed and counted in five random fields using an inverted microscope.

### 2.6. Lactate Production Detection and Glucose Consumption Assay

Equal numbers (1 × 10^6^) of NSCLC cells were seeded in 6-well plates. After treatment with 40 *μ*g/ml Rh2 for 48 h, the cell culture supernatant was collected to measure the lactate and glucose production by using the Lactate Assay kit (JianCheng Bioengineering Institute, Nanjing, China) and the Glucose Assay kit (Wanlei-bio, Shenyang, China), respectively, according to the manufacturer's instructions. Lactate production and glucose consumption were calculated as follows:

  Lactate production (mM) = (sample OD – blank OD/standard OD – blank OD) × standard concentration (3 mM) × dilution ratio (5 × in this study)
  Glucose production (mM) =  blank OD or sample OD/standard OD × standard concentration (5.55 mM)
  Glucose consumption (mM) = blank glucose production−sample glucose production

### 2.7. Western Blotting and Nuclear and Cytoplasm Isolation

Total proteins from NSCLC cells with or without Rh2 treatment were lysed in RIPA (Beyotime) lysis buffer supplemented with 1% phenylmethanesulfonyl fluoride. 40 *μ*g proteins were separated using 10% SDS-PAGE, transferred onto 0.45 *μ*m polyvinylidene fluoride membranes (Millipore), and incubated overnight at 4°C with primary antibodies (Table 1), followed by incubating with a horseradish peroxidase-conjugated anti-rabbit as previously described [9]. ImageJ software was used to evaluate the gray value of each band.

For isolating the nuclear and cytoplasm cell fractions, a Nuclear and Cytoplasmic Protein Extraction kit (Beyotime) was used. The collected cells were suspended in ice-cold hypotonic buffer and incubated on ice for 20 min. The extracts were then centrifuged at 12,000 × g for 5 min, and the supernatants were collected as cytosolic fractions. The pellets were washed with ice-cold PBS and resuspended in lysis buffer, followed by vortexing at the highest speed for 30 min. These extracts were centrifuged at 12,000 × g for 10 min, and the supernatants were collected as the nuclear fractions.

### 2.8. Immunofluorescence Staining

Cells were seeded in 20 mm culture plates and cocultured with or without Rh2 for 24 h, then washed with PBS, fixed with 4% paraformaldehyde for 15 min, and permeabilized in 0.1% TritonX-100 for 5 min. After blocking with 5% bovine serum albumin for 1 h at room temperature, the cells were incubated with primary antibody against p-STAT3 (dilution 1 : 100) overnight at 4°C. Then, FITC-conjugated secondary antibody (dilution 1 : 200) was incubated with the cells for 1 h in the dark at room temperature, and the cells were stained with 4′,6-diamidino-2-phenylindole for 5 min. Images were captured using a fluorescence microscope.

### 2.9. Statistical Analysis

All experiments were repeated at least three times. Data were analyzed using GraphPad Prism 8.0 software and presented as mean ± standard deviation. Student's *t*-test and one-way ANOVA were used to compare the differences between two groups or multiple groups, respectively. *P* < 0.05 was considered statistically significant.

## 3. Results

### 3.1. Rh2 Suppressed the Growth of NSCLC Cells

**Figure 1:**
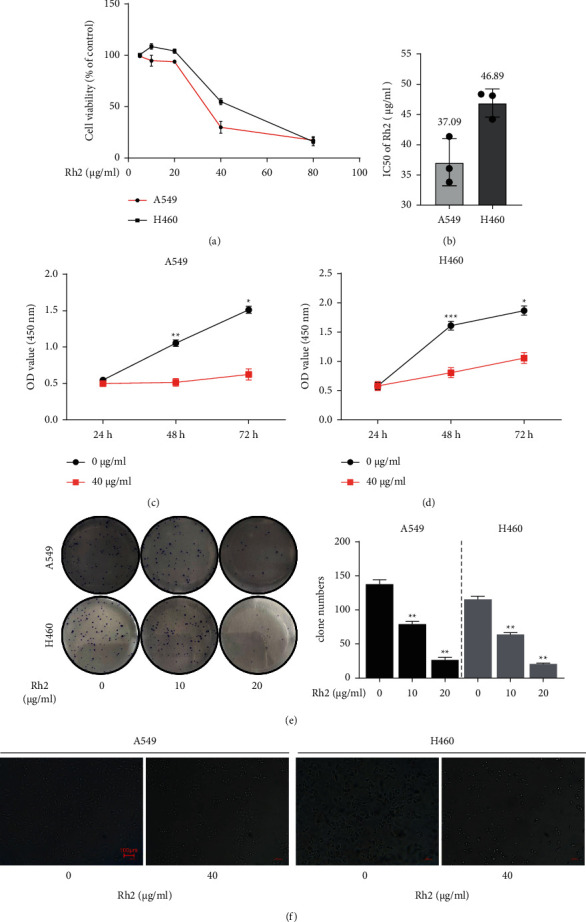
Rh2 inhibited NSCLC cells proliferation. (a) Cell viability of A549 and H460 cell lines was detected using CCK-8 assay. (b) The IC50 values of Rh2 for 48 h in A549 and H460 cell lines. (c, d) The NSCLC cells were treated with 40 *μ*g/ml Rh2 for different time intervals (24, 48, and 72 h). The cell viability was detected by CCK-8 and presented as absorbance value (OD). (e) Colony formation assay of NSCLC cells. Clone numbers were counted using a microscope. (f) The morphology changes of NSCLC cells induced by 40 *μ*g/ml Rh2 were observed using a light microscope (10x). Scale bar = 100 *μ*m. Data are shown as mean ± SD of three independent experiments. ^*∗*^*P* < 0.05, ^*∗∗*^*P* < 0.01, and ^*∗∗∗*^*P* < 0.001 vs. control groups.

First of all, A549 and H460 cell lines were treated with different concentrations of Rh2 for 48 h to access their IC50 values. The cell viability decreased as the drug concentration increased (Figure 1(a)), with IC50 values of (37.09 ± 3.88) and (46.89 ± 2.32) *μ*g/ml, respectively (Figure 1(b)). The IC50 dose, i.e., approximately 40 *μ*g/ml Rh2, was chosen for the subsequent experiments. Then, we also observed that NSCLC cell growth was inhibited by Rh2 in a time-dependent manner (Figures 1(c) and 1(d)). The clone numbers of NSCLC cells were significant lower in Rh2-treated groups than those in the control groups (Figure 1(e)) and the inhibition effect was concentration dependent. In addition, cell morphology changes induced by Rh2 were observed directly by using a microscope. As shown in Figure 1(f), after treated with Rh2, the number of cells was reduced, and the cell shape was smaller and more irregular compared to the control groups; moreover, the cells took on indistinct margins and looser intercellular connections, compared to the control group. These morphology changes were in line with the effects of Rh2 on other tumor cells [10]. Collectively, the abovementioned results suggest that Rh2 could suppress NSCLC cell growth in both a concentration- and time-dependent manner.

### 3.2. Rh2 Increased Apoptosis Level in NSCLC Cells

**Figure 2:**
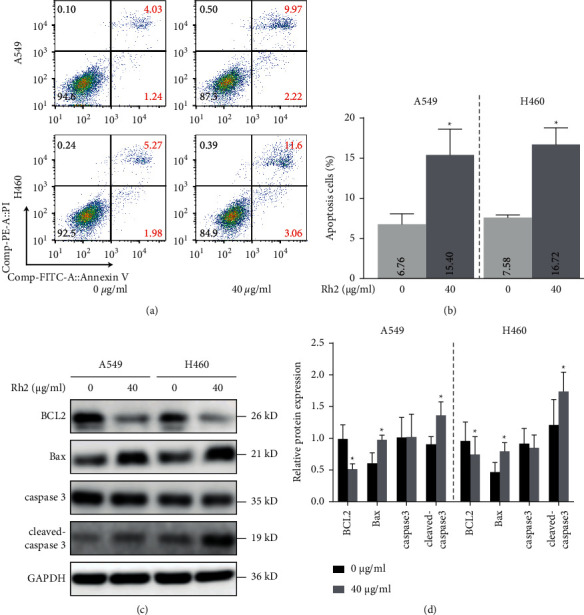
Rh2 induced apoptosis in NSCLC cells. (a) After treatment of A549 and H460 cells without or with 40 *μ*g/ml Rh2 for 48 h, cells were stained with Annexin V-FITC/PI and detected by using a flow cytometer. (b) The quantitative analysis of the apoptotic cell percentages is shown. (c) After treatment with 40 *μ*g/ml Rh2 for 48 h, the protein levels of BCL2, Bax, caspase 3, and cleaved caspase 3 were determined by western blotting. (d) The relative western blot gray values are shown in the histogram. Data are shown as mean ± SD of three independent experiments. ^*∗*^*P* < 0.05 vs. control groups.

Since the cell morphology change induced by Rh2 was similar to apoptosis, we supposed that the potential mechanism underlying the antitumor effect of Rh2 was increasing apoptosis level in NSCLC cells. After treatment with 40 *μ*g/ml Rh2 for 48 h, the percentage of apoptotic cells was significantly higher than that in the control group (Figures 2(a) and 2(b)). In addition, the expression of mitochondrial apoptotic-related proteins was regulated by Rh2. Antiapoptosis protein BCL2 was downregulated, while proapoptosis proteins Bax and cleaved caspase 3 were upregulated after Rh2 treatment (Figures 2(c) and 2(d)). These results indicated that Rh2 could increase apoptosis level in NSCLC cells via regulating the mitochondrial apoptotic way.

### 3.3. Rh2 Inhibited NSCLC Cell Invasion and Migration

**Figure 3:**
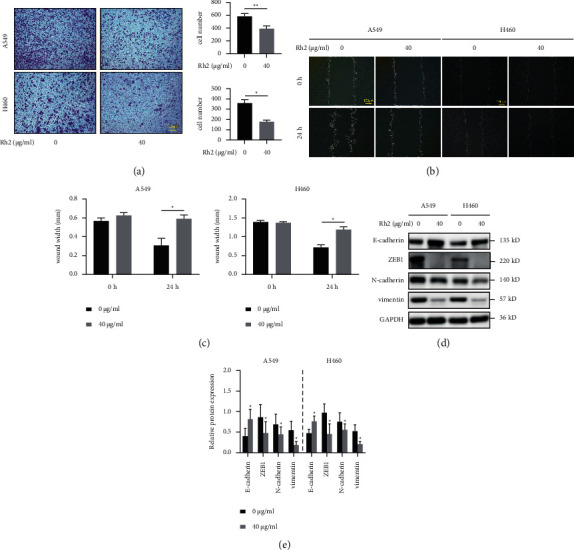
Rh2 inhibited NSCLC cell invasion and migration. (a) The invasion of A549 and H460 cells after treatment without or with 40 *μ*g/ml Rh2 for 24 h was assessed using transwell assays. The invaded cells were stained, and the cell numbers were counted by ImageJ software. (b) The migration of A549 and H460 cells after treatment without or with 40 *μ*g/ml Rh2 for 24 h was detected using wound healing assays. (c) The gap distance was measured and calculated by ImageJ software. Wound width (mm) = wound area/wound length. (d) After treatment with 40 *μ*g/ml Rh2 for 48 h, the protein levels of the EMT markers E-cadherin, ZEB1, N-cadherin, and vimentin in NSCLC cells were determined by western blotting. (e) The relative western blot gray values are shown in the histogram. Data are shown as mean ± SD of three independent experiments. ^*∗*^*P* < 0.05, ^*∗∗*^*P* < 0.01 vs. control groups.

Suppressing tumor cell migration and invasion has been confirmed as a successful strategy in cancer treatment. It was reported that Rh2 could suppress these processes in many tumor cells including NSCLC. Firstly, we confirmed that NSCLC cell invasion ability was significantly delayed under Rh2 treatment (Figure 3(a)). Similarly, the wound healing assay suggested that the migration ability of Rh2-treated NSCLC cells was also significantly decreased (Figures 3(b) and 3(c)). Since the epithelial-mesenchymal transition (EMT) process is a key regulator of tumor metastasis, we performed western blotting to explore whether Rh2 has an effect on EMT-associated markers in NSCLC cells. After Rh2 treatment, the level of the epithelial marker E-cadherin increased, whereas the levels of E-cadherin-binding protein ZEB1 and the mesenchymal markers N-cadherin and vimentin decreased (Figures 3(d) and 3(e)). Thus, we concluded that Rh2 inhibited NSCLC cell invasion and migration mainly via suppressing EMT.

### 3.4. Rh2 Induced a Metabolic Shift in NSCLC Cells

**Figure 4:**
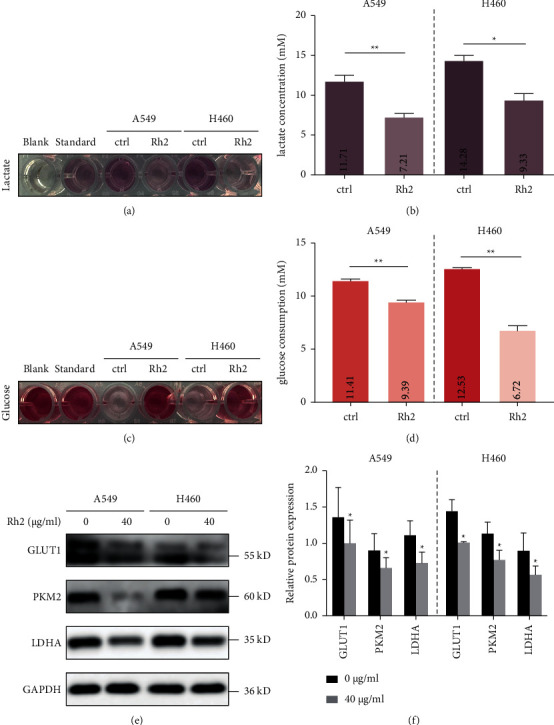
Rh2 induced a metabolic shift in NSCLC cells. A549 and H460 cells were treated without or with 40 *μ*g/ml Rh2 for 48 h. (a) The color depth of lactate test solution was evaluated visually. Blank: phosphate buffered saline without cells. Standard (lactate concentration is 3 mM): solution provided in the assay kit. (b) The lactate concentrations of control and Rh2 groups were calculated according to the formula described in the methods section. (c) The color depth of glucose test solution was evaluated visually. Blank: the culture medium (RPMI-1640) without cells. Standard (glucose concentration is 5.55 mM): solution provided in the assay kit. (d) The glucose consumptions of control and Rh2 groups were calculated according to the formula described in the methods section. (e) The protein levels of metabolic enzymes GLUT1, PKM2, and LDHA were tested by western blotting. (f) The relative western blot gray values are shown in the histogram. Data are shown as mean ± SD of three independent experiments. ^*∗*^*P* < 0.05, ^*∗∗*^*P* < 0.01 vs. control groups.

As is well known, the Warburg effect, which is characterized by increased glycolysis and lactate production regardless of oxygen availability, is a novel landmark of tumor. However, whether Rh2 is capable of impacting tumor metabolic status remains unknown. Therefore, we investigated the effect of Rh2 on lactate secretion and glucose consumption of NSCLC cells. As shown in Figure 4(a), the color of the lactate test solution in the Rh2 group was lighter than that of the control group, which indicated that the lactate concentration in the Rh2-treated cell culture medium was significantly lower than that of the control group (Figure 4(b)). On the contrary, the color of the glucose test solution in the Rh2 group was darker than that in the control group (Figure 4(c)), which meant Rh2 suppressed glucose uptake of NSCLC cells (Figure 4(d)). Then, the expression of key glycolysis-related metabolic enzymes was detected. The level of GLUT1, which is an important glucose transporter, was decreased in Rh2-treated cells. Besides, the expressions of PKM2 and LDHA, which function as catalysts for pyruvate and lactic acid production, respectively, were both inhibited by Rh2. These results revealed that Rh2 was able to induce a metabolic shift in NSCLC cells by regulating key enzymes in the glycolysis process.

### 3.5. Rh2 Inhibited Glycolysis of NSCLC Cells by Suppressing STAT3/C-Myc Axis Activity

**Figure 5:**
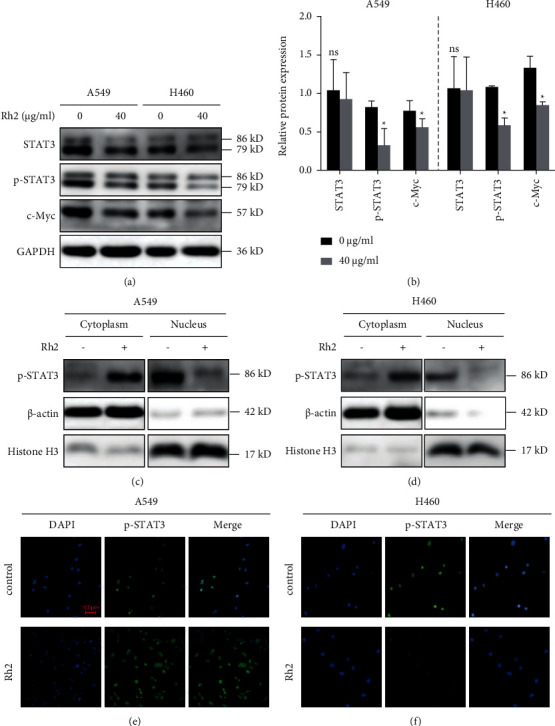
Rh2 induced metabolic shift via suppressing STAT3/c-Myc axis activity. (a) A549 and H460 cells were treated without or with 40 *μ*g/ml Rh2 for 48 h. The protein levels of STAT3, p-STAT3 (Tyr 705), and c-Myc were tested by western blotting. (b) The relative western blot gray values are shown in the histogram. (c, d) Rh2 inhibited the nuclear translocation of p-STAT3 by western blot after nuclear and cytoplasm isolation. *β*-Actin and Histone H3 were used as cytoplasm and nuclear control, respectively. (e, f) Rh2 inhibited the nuclear translocation of p-STAT3 by immunofluorescence staining (10×). Scale bar = 100 *μ*m. ^*∗*^*P* < 0.05 vs. control groups. ns, not significant.

\In addition to tumorigenesis, STAT3 and c-Myc play important roles in facilitating tumor cell glycolysis [11, 12]. Therefore, we investigated the potential effect of Rh2 on the STAT3/c-Myc axis. The results showed that the expression of STAT3 remained unchanged, while its phosphorylation level (p-STAT3) and c-Myc were decreased after Rh2 treatment (Figure 5(a) and 5(b)). In addition, since the nuclear translocation of p-STAT3 was recognized as a marker of its activation, the nuclear and cytoplasm isolation assay was performed. The results showed that, after Rh2 treatment, the expression of p-STAT3 in the cytoplasm was increased, while the opposite results were observed in the nuclear (Figure 5(c) and 5(d)). Furthermore, the immunofluorescence staining was also used to confirm the effect of Rh2 inhibiting the nuclear translocation of p-STAT3 (Figure 5(e) and 5(f)). Collectively, these results indicated that Rh2 induced a metabolic shift through suppressing STAT3/c-Myc axis activity.

### 3.6. STAT3/C-Myc Axis Is Crucial for Rh2-Induced Metabolic Shift

**Figure 6:**
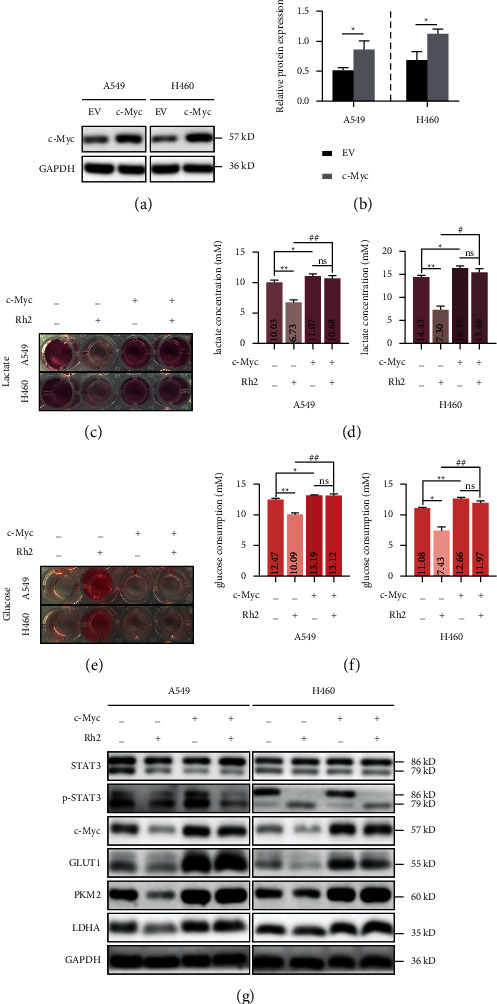
The inhibitory effect of Rh2 on glycolysis was dependent on the STAT3/c-Myc axis. (a) A549 and H460 cells were transfected with c-Myc expression plasmid for 48 h. The transfection efficiency was detected by western blot, and (b) the relative gray values were shown in a histogram. (c) The color depth of lactate test solution in each group was evaluated visually. (d) The lactate concentration of each group was calculated. (e) The color depth of glucose test solution in each group was evaluated visually. (f) The glucose consumption of each group was calculated. (g) The expression levels of key metabolic enzymes under different treatments were detected by western blotting. Data are shown as mean ± SD of three independent experiments. ^*∗*^*P* < 0.05, ^*∗∗*^*P* < 0.01 vs. control groups. ^#^*P* < 0.05, ^##^*P* < 0.01. ns, not significant. EV, empty vector.

To further demonstrate whether the effect of Rh2-induced metabolic shift in NSCLC cells was STAT3/c-Myc axis dependent, c-Myc expression vector and empty vector were ectopically transfected into A549 and H460 cells for 48 h. The transfection efficiency was detected by western blot (Figures 6(a), 6(b)). As expected, overexpression of c-Myc significantly promoted the lactate secretion (Figures 6(c) and 6(d)) and glucose consumption (Figures 6(e) and 6(f)) of NSCLC cells. Interestingly, the expression of STAT3 or p-STAT3 was not affected by the expression change of c-Myc, which indicated that STAT3 is a potential upstream molecule of c-Myc (Figure 6(g)). In addition, the inhibition of Rh2 on the glycolysis process and the levels of key metabolic enzymes was abolished upon c-Myc overexpression (Figures 6(c)–6(g)). Overall, these results revealed that the STAT3/c-Myc axis is crucial for the metabolic shift regulated by Rh2 in NSCLC cells.

## 4. Discussion

**Figure 7:**
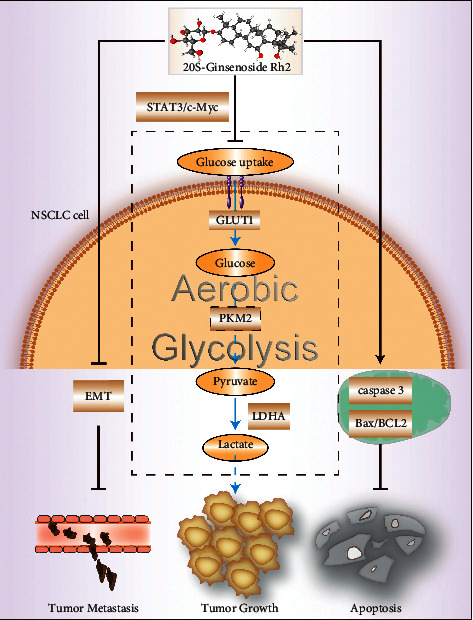
Schematic representation of the antitumor effect of Rh2 in NSCLC cells. Rh2 inhibited NSCLC cell invasion and migration by suppressing EMT and promoted apoptosis through regulating the mitochondrial apoptotic way. The metabolic shift induced by Rh2 in NSCLC depended on regulating the expression of glycolysis-related enzymes via the STAT3/c-Myc axis.

The tumor cells take advantage of the Warburg effect to not only meet their high energy demands but also form an acidic microenvironment to support their survival and malignant phenotype. Although emerging evidence has indicated that Rh2 exerts a potent antitumor effect on diverse tumor cells, its role in regulating glycolysis has not been clarified. In the present study, we confirmed that Rh2 exhibits inhibition of proliferation, invasion, and migration in NSCLC cells and demonstrated that Rh2 suppresses aerobic glycolysis of NSCLC cells via the STAT3/c-Myc axis (Figure 7).

Firstly, the effect of Rh2 on the proliferation and cloning capacity of A549 and H460 cells has been verified. The results showed that the proliferation inhibition effect of Rh2 on NSCLC cells was time and concentration dependent. In addition, we further observed that Rh2 could significantly promote apoptosis by downregulating the expression of antiapoptotic protein BCL2 and upregulating the expression of proapoptotic protein Bax and cleaved caspase 3. These observations are consistent with the effects of Rh2 reported in other tumors, such as cervical cancer [13], osteosarcoma [14], and prostate cancer [15]. Then, we verified that Rh2 could suppress the invasion and metastasis ability of NSCLC cells through regulating the EMT process, in line with the role of Rh2 in endometrial cancer [16], cervical cancer [17], and colorectal carcinoma cells [18]. In addition, it has been reported that the antitumor properties of Rh2 were related to its structure [19]. Compared with 20(R)-Rh2, 20(S)-Rh2 showed stronger cytotoxicity to the proliferation of prostate cancer and NSCLC cells [20, 21], indicating the stereochemistry of hydroxylation at the carbon-20 of Rh2 was important for its cytotoxicity. Besides, molecular docking technology demonstrated that Rh2 had a high affinity with epithelial cell adhesion molecule, which was identified as a potential therapy target in esophageal carcinoma, through establishing hydrogen bonding with Leu240 [22]. However, it was not available here to clarify which region of STAT3 or c-Myc is the docking mode with Rh2; hence, further investigations are needed.

Glucose is a raw material for glycolysis, whereas lactate is an important final product of glycolysis. The concentration of lactate in the culture medium and the consumption of glucose could represent the level of glycolysis of NSCLC cells. It was reported that another subtype of ginsenoside, Rg3, could inhibit lactate production in ovarian cancer cells [23], and similarly, we observed that Rh2 could significantly reduce the level of lactate produced by NSCLC cells and decrease their uptake of glucose. In addition, the expression of GLUT1, which is an important glucose transporter [24], was decreased after Rh2 treatment. PKM2, one of the key enzymes in the glycolysis process, is involved in the production of pyruvate [25]. LDHA functions as a key enzyme in the last step of the glycolysis pathway and catalyzes the conversion of pyruvate to lactate [26]. We found that Rh2 treatment significantly inhibited the expression of these enzymes, suggesting Rh2 could decrease the glycolysis level partly through regulating glycolysis-related enzymes. Similar glycolysis suppression function was observed in other kinds of ginsenosides. For example, Rg3 inhibited the Warburg effect in ovarian cancer cells and gliomas via targeting microRNA or long noncoding RNA [23, 27]. Furthermore, it has been reported that STAT3 acts as a transcription factor to promote the expression of hexosaccharide kinase 2, which is a major glycolysis rate-limiting enzyme, resulting in glycolysis promotion [11] and c-Myc could also participate in glutamate metabolism to catalyze the decomposition of glutamine to produce lactate [12]. Thus, the STAT3/c-Myc axis plays crucial roles in glycolysis regulation. For example, the STAT3/c-Myc axis regulates the energy metabolism of gastric cancer cells by synergistically communicating with the mTOR/PKM2 pathway [28]. In the present study, we found that the protein levels of p-STAT3 and c-Myc were decreased, and the nuclear translocation of p-STAT3 was inhibited upon Rh2 treatment, suggesting a decreased activity of this axis. Interestingly, the inhibition effect of Rh2 on the glycolysis process of NSCLC cells was abolished by overexpressing c-Myc, which meant the STAT3/c-Myc axis was crucial for Rh2-induced metabolic shift.

However, the influence factors of the glycolysis process are more complex than we have known, such as PI3K/AKT/mTOR signaling [29], and noncoding RNAs, including microRNAs, long noncoding RNAs, and circular RNAs [30], are all involved in this process. Moreover, the activity of these signaling factors can be regulated by protein posttranslational modifications, such as ubiquitinated or deubiquitinated [31]. Thus, whether Rh2 plays roles in regulating these factors remains to be elucidated.

## 5. Conclusions

Collectively, we confirmed that Rh2 inhibited the proliferation and metastasis of NSCLC cells by promoting apoptosis and suppressing EMT, respectively. Notably, Rh2 exerted a glycolysis inhibition effect through the STAT3/c-Myc axis in NSCLC. This novel regulatory role of Rh2 provides a new perspective for NSCLC treatment. Rh2 could be used as a tumor energy blocker and the combination of Rh2 with an STAT3 or c-Myc inhibitor may be a promising therapeutic approach for patients with NSCLC. However, further animal studies or clinical trials need to be performed to confirm these.

## Figures and Tables

**Table 1:** List of antibodies used in the study.

Target	Company	Catalog number	Host	Dilution
STAT3	Cell Signaling Technology	4904S	Rabbit	1 : 1000
p-STAT3 (Tyr705)	Cell Signaling Technology	9131S	Rabbit	1 : 1000
c-Myc	Cell Signaling Technology	9402S	Rabbit	1 : 1000
PKM2	Cell Signaling Technology	3198S	Rabbit	1 : 1000
LDHA	Wanlei-bio	WL03271	Rabbit	1 : 1000
GLUT1	Wanlei-bio	WL01163	Rabbit	1 : 1000
E-cadherin	Cell Signaling Technology	3195S	Rabbit	1 : 500
N-cadherin	ZEN-Bio (Chengdu, China)	380671	Rabbit	1 : 1000
ZEB1	Cell Signaling Technology	70512S	Rabbit	1 : 1000
Vimentin	Cell Signaling Technology	5741S	Rabbit	1 : 1000
Bcl-2	Cell Signaling Technology	15071S	Rabbit	1 : 1000
Bax	Cell Signaling Technology	2774S	Rabbit	1 : 1000
Caspase-3	Cell Signaling Technology	14220S	Rabbit	1 : 1000
Histone H3	Cell Signaling Technology	4499S	Rabbit	1 : 1000
*β*-Actin	Wanlei-bio	WL01372	Rabbit	1 : 1000
GAPDH	Wanlei-bio	WL03412	Rabbit	1 : 5000

## Data Availability

The data used to support the findings of this study are available from the corresponding author upon request.
